# Annotations

**Published:** 1894-08-04

**Authors:** 


### Annotations.

### Curiosities of American Lunacy.

The delusions of the insane are innumerable. It is very seldom that a lunatic starts anything novel by way of delusion. The number of insane persons who have been made of glass, as they thought, and would break if they were touched, is legion. So, too, the princes and princesses who throng the world's lunatic asylums are more than sufficient to furnish all the thrones in both hemispheres ten times over. At Chicago the other day Dr. Brower illustrated a lecture on mental diseases, and showed one or two patients whose delusions might almost be considered novel. One poor woman, for example, thought her head was full of the letters of the alphabet, and that they would fall out and bring about some tremendous calamity if she began to eat. Accordingly she refused all food. A man was under the impression that his brain was tenanted by rabbits and squirrels, and in accordance with the frisky habits of those light-hearted rodents he was in a constant state of brimming excitement and laughter. Whilst in Europe one expects the "grand" lunatic to be a Prince or an Emperor, in America one naturally looks for the delusion of grandeur to take the "millionaire" form. And the facts justify this natural guess. Dr. Brower showed his students a poor woman who was quite the greatest personage in the asylum in her own estimation. Two million dollars was the smallest fortune she would admit to being the possessor of. She was, as Dr. Broner said, of very "blue blood" too, inasmuch as she claimed descent from Henry Clay. But even she, rich as she was, could not equal a male lunatic who was shown a few minutes later. Three million dollars, he declared, was the sum of his earthly possessions, and he very magnificently offered to take the doctor "round the world in his own craft"—a truly transatlantic display of generosity. These cases, like every case of lunacy, all go to prove that the lunatic is but the natural man exaggerated, and no longer controlled by King Reason. Dr. Brower incidentally showed also, what we have often contended here, that lunacy is one of the most preventable of all diseases; and that if medicine could govern the world for a century or two, instead of being its very humble servant, a very different face would be put upon human affairs generally.

### Playgrounds for Little Players.

The Earl of Meath told the members of the British Institute of Public Health, at their annual congress the other day, that London possesses 3,090 acres of public pleasure grounds, in parcels of not less than ten acres apiece. Of gardens and other plots of ground smaller than ten acres the name is "legion." This acreage is vast, and we are thankful for it. But when one begins to apply figures to it, and to divide the number of children in the metropolis by the number of acres of available playground, one cannot help saying, as certain surprised early Christians said of the loaves and fishes, "What are they among so many"? In most cities the proportion of children and young people to adults is as four to two. If we assume that in London the proportion is no more than three to two we shall have the enormous total of 3,000,000 children and young people for the whole metropolis. This gives us the large number of 1,000 children for every single acre of available playground. There is then left no recreation space at all for the two millions of adults which remain. Besides, how could a thousand children play on an acre of ground? To those who realise what an acre is, it presents the appearance of a moderate-sized kitchen garden, or of a lawn tennis ground which will accommodate three or four courts. It is much too small for cricket, four or five acres being the least space on which that English game can be comfortably played. So that if we had over 1,000 children on each acre of London's playground at any one time they would look much more like packed sardines than playing children. The Earl of Meath asks for more ground. He desires also that all our suitable open spaces should be lighted by electricity, in order that they may be available during the long winter evenings, and so get young people out of their stuffy little rooms into the open air. Finally, he insists that municipal authorities ought to be empowered to provide winter gardens and other recreation appliances for the good of the people. Now these are extremely practical and valuable suggestions, and they ought to become actualities at once. Indeed, they ought to have become actualities long ago. If newspaper writers knew a little more science, and had a little more faith in it, they would very speedily bring about the realisation of Lord Meath's hopes.

### An Outrage at the British Medical Association.

The proceedings at the first general meeting of the members of the British Medical Association on Tuesday were outrageous, and cast a grave reflection upon the members as a whole. It is all very well to say that it is amusing to listen to a travesty of criticism served up hot by a crank of the first water. But the members owed it to themselves to publicly resent and condemn to silence anyone who indulged in so outrageous and unjustifiable a personal attack as that sprung upon the Editor of its journal. No doubt Mr. Ernest Hart is quite able to defend himself, but the honour of the Editor should very nearly concern the honour of each and all of the members of the British Medical Association. Criticism well conceived and fairly uttered is always to be welcomed by honest men desirous of securing the highest ends; but the vapourings of irresponsible and ill-informed persons of no particular position or authority are quite another matter. Mr. Hart has displayed erudition, courage, and great self-denial in the conduct of the *British Medical Journal*. His conspicuous success as an editor is proved by the present position of the association, with its more than fifteen thousand members, and by the circumstance that as a medical journalist he is admittedly *facile princeps* amongst his contemporaries. He has made signally few mistakes, whilst his achievements have brought him fame and deservedly great influence and power. Where would the British Medical Association stand to-day without its journal, and who but Mr. Ernest Hart has made the *British Medical Journal* what it is to-day? By all means let there be honest differences of opinion; but when abuse descends to the level of a ridiculous and unjustifiable attack like that to which Mr. Hart was subjected at Bristol, we had a right to expect that a wave of justifiable indignation would have swept the offender from the platform.

